# Hydrogen-rich water supplementation promotes muscle recovery after two strenuous training sessions performed on the same day in elite fin swimmers: randomized, double-blind, placebo-controlled, crossover trial

**DOI:** 10.3389/fphys.2024.1321160

**Published:** 2024-04-12

**Authors:** Barbora Sládečková, Michal Botek, Jakub Krejčí, Michal Valenta, Andrew McKune, Filip Neuls, Iva Klimešová

**Affiliations:** ^1^ Department of Social Sciences in Kinanthropology, Faculty of Physical Culture, Palacký University Olomouc, Olomouc, Czechia; ^2^ Department of Natural Sciences in Kinanthropology, Faculty of Physical Culture, Palacký University Olomouc, Olomouc, Czechia; ^3^ Department of Sport, Faculty of Physical Culture, Palacký University Olomouc, Olomouc, Czechia; ^4^ Faculty of Health, UC-Research Institute for Sport and Exercise, University of Canberra, Canberra, NSW, Australia; ^5^ Discipline of Biokinetics, Exercise and Leisure Sciences, School of Health Sciences, University of KwaZulu-Natal, Durban, South Africa

**Keywords:** molecular hydrogen, creatine kinase, exercise-induced muscle damage, exercise, muscle pain, peripheral fatigue

## Abstract

**Purpose:** Molecular hydrogen has been shown to possess antioxidant, anti-inflammatory, ergogenic, and recovery-enhancing effects. This study aimed to assess the effect of molecular hydrogen administration on muscle performance, damage, and perception of soreness up to 24 h of recovery after two strenuous training sessions performed on the same day in elite fin swimmers.

**Methods:** Eight females (mean ± SD; age 21.5 ± 5.0 years, maximal oxygen consumption 45.0 ± 2.5 mL.kg^−1^.min^−1^) and four males (age 18.9 ± 1.3 years, maximal oxygen consumption 52.2 ± 1.7 mL.kg^−1^.min^−1^) performed 12 × 50 m sprints in the morning session and a 400 m competitive performance in the afternoon session. Participants consumed hydrogen-rich water (HRW) or placebo 3 days before the sessions (1,260 mL/day) and 2,520 mL on the experimental day. Muscle performance (countermovement jump), muscle damage (creatine kinase), and muscle soreness (100 mm visual analogue scale) were measured during the experimental day and at 12 and 24 h after the afternoon session.

**Results:** HRW compared to placebo reduced blood activity of creatine kinase (156 ± 63 vs. 190 ± 64 U.L^−1^, *p* = 0.043), muscle soreness perception (34 ± 12 vs. 42 ± 12 mm, *p* = 0.045), and improved countermovement jump height (30.7 ± 5.5 cm vs. 29.8 ± 5.8 cm, *p* = 0.014) at 12 h after the afternoon session.

**Conclusion:** Four days of HRW supplementation is a promising hydration strategy for promoting muscle recovery after two strenuous training sessions performed on the same day in elite fin swimmers.

**Clinical Trial Registration:** clinicaltrials.gov, identifier NCT05799911

## 1 Introduction

Participation in fin swimming has increased worldwide. Fin swimming is metabolically demanding and this is particularly due to using a specialized fin(s) that propels the swimmer at great speeds (Wang et al., 2012). Well-trained fin swimmers, similarly to non-fin swimmers, perform two water-training sessions a day (Stavrou et al., 2019), including high-intensity interval training (HIIT) (Aspenes and Karlsen, 2012; Budnik-Przybylska et al., 2018), which typically involves repeated high-intensity short intervals interspersed with active or passive recovery (Buchheit and Laursen, 2013). HIIT is a very popular training method, however it is associated with exercise-induced muscle damage (EIMD), evidenced by increased protein such as creatine kinase (CK), myoglobin, and lactate dehydrogenase in the bloodstream together with elevated muscle pain manifesting immediately post-exercise to several days after HIIT (Leite et al., 2023). Moreover, during high-intensity exercise such as repeated sprinting, formation of excessive reactive oxygen and nitrogen species may cause exercise-induced oxidative damage to cellular structures and mitochondrial fatigue (Calbet et al., 2020). The origin of EIMD is linked with metabolic and/or mechanical stress, including oxidative stress and inflammation (Pyne, 1994; Peake et al., 2017). The severity of the EIMD depends on the type, intensity, and duration of the exercise (Tee et al., 2007), as well as on individual training status (Brancaccio et al., 2010). Further, EIMD has been associated with the transient loss of muscle strength and power, swelling, and delayed onset muscle soreness (DOMS) (Peake et al., 2017), which typically peaks 24–48 h after strenuous or unaccustomed exercise (Cheung et al., 2003). Traditionally, impairment in lower limb muscle function due to EIMD has been assessed via the measurement of countermovement jump (CMJ) height (Markovic et al., 2004).

Exercise-induced muscle pain can be noninvasively evaluated using psychometric tools such as the visual analogue scale (VAS) (Heller et al., 2016), while the activity of blood CK is widely accepted as an indirect biomarker of both EIMD and changes in membrane permeability (Brancaccio et al., 2010) that involves both connective tissue and muscle cells (Mougios, 2007; Baird et al., 2012; Peake et al., 2017). The VAS is the most frequently used and reliable, non-invasive method for the evaluation of pain severity and relief (Price et al., 1983; Boonstra et al., 2008). Furthermore (Kawamura et al., 2018), found a significant correlation at 24 and 48 h after exercise between VAS, CK, and plasma pro-inflammatory interleukin-6 concentration. However, correlations between VAS and blood CK activity are not consistent (Hartmann and Mester, 2000). In contrast to other sports, swimming generally involves mainly non-weight-bearing activities and concentric contractions of the upper and lower limb muscles that result in minor muscle damage and only a small increase in the activity of blood CK (Mougios, 2007). Despite this fact, some studies have reported a significant increase in blood CK activity immediately after (Deminice et al., 2010), at 1 h (Tauler et al., 2008), and at 24 h after strenuous swimming (Rahmanian et al., 2022).

Molecular hydrogen (H_2_) is suggested as a healthy and safe gas (Nicolson et al., 2016; Cole et al., 2022; Salomez-Ihl et al., 2024) with a potent antioxidant effect (Ohsawa et al., 2007). In addition to its antioxidant properties, H_2_ has been found to have anti-inflammatory properties (Gharib et al., 2001), antiapoptotic properties (Nicolson et al., 2016), and properties that modulate signal transduction and gene expression (Ohta, 2014; Slezak et al., 2021). Due to its health benefits (Ohta, 2014; Ichihara et al., 2015; Botek et al., 2022b; Johnsen et al., 2023), supplementation with H_2_ has become popular among athletes to enhance performance and reduce fatigue (LeBaron et al., 2019; Botek et al., 2020; Kawamura et al., 2020; Shibayama et al., 2020; Botek et al., 2021; Timón et al., 2021; Botek et al., 2022a; Jebabli et al., 2023; Zhou et al., 2023). Several recent studies demonstrated that H_2_ reduces exercise-induced pro-inflammatory response and oxidative stress (Ara et al., 2018; Nogueira and Branco, 2021), blood lactate concentrations and improves muscle function (Aoki et al., 2012; Botek et al., 2021). In addition to its physiological benefits, H_2_ may also reduce perception of muscle soreness after a single-strength training session (Todorovic et al., 2020; Botek et al., 2021; Yoshimura et al., 2023) and after down-hill running (Kawamura et al., 2016). However, there is still limited research examining the effects of hydrogen-rich water (HRW) supplementation on muscle damage and perception of muscle soreness within and after two physically highly demanding exercise sessions performed on the same day during routine training periodization in elite fin swimmers.

Therefore, the primary objective of this study was to evaluate the effect of HRW ingestion on muscle function, damage, and soreness perception up to 24 h of recovery after two strenuous training sessions performed on the same day in elite fin swimmers.

## 2 Materials and methods

### 2.1 Participants

**FIGURE 1:**
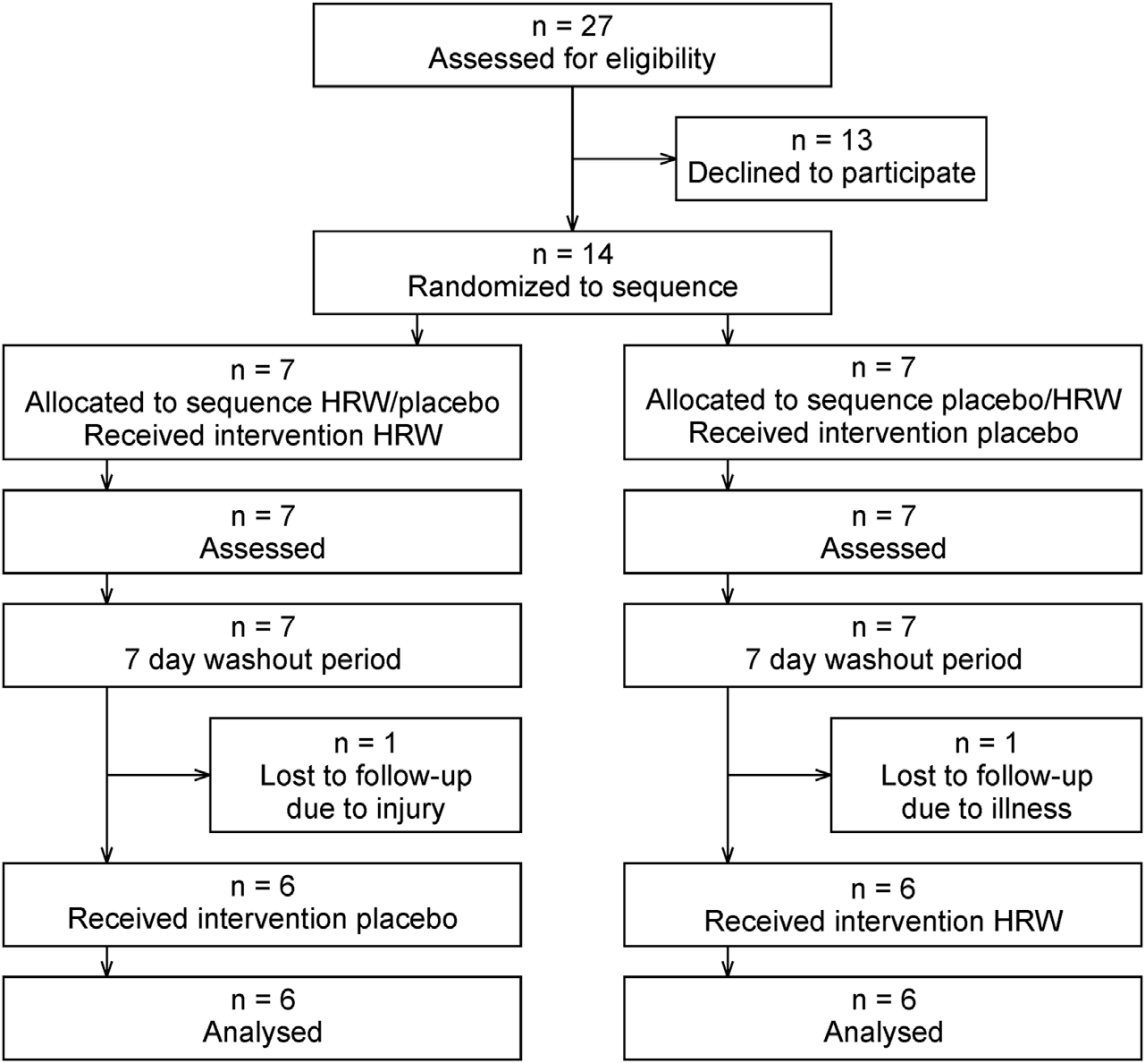
CONSORT flow diagram. HRW, hydrogen-rich water.

Fourteen national and international elite Czech fin swimmers participated in the study. Two swimmers did not complete the protocol due to injury (one participant) and illness (one participant), with the final population consisting of 12 participants (eight females and four males, Figure 1). The characteristics of the participants are shown in Table 1. The study was carried out according to the Declaration of Helsinki and was approved by the Ethics Committee of the Faculty of Physical Culture, Palacký University Olomouc (reference number 11/2023). Participation in the study was voluntary and written informed consent was provided after detailed verbal explanation of the objectives and protocol. Participants were requested to not take any supplements at least 2 weeks before the experiment and to avoid strenuous exercise in the 24 h before the first morning session. In addition to HRW/placebo supplementation, a dose of 0.5–1.0 L of tap water was recommended during the simulated racing day. During the recovery on the following day, participants were requested to keep hydrated, drinking between 1.5 and 2.5 L of tap water.

**TABLE 1:** Characteristics of the fin swimmers.

	Female	Male	*p*
Variable	Mean ± SD	Mean ± SD	
Sample size	8	4	
Age (years)	21.5 ± 5.0	18.9 ± 1.3	0.72
Body height (cm)	164 ± 6	184 ± 9	0.004
Body mass (kg)	62 ± 7	78 ± 10	0.012
BMI (kg.m^−2^)	22.8 ± 1.2	23.1 ± 1.9	0.81
Body fat (%)	22.0 ± 1.5	12.1 ± 4.6	0.004
VO_2_max (mL.kg^−1^.min^−1^)	45.0 ± 2.5	52.2 ± 1.7	0.004

SD, standard deviation; *p*, statistical significance of the comparison between males and females (Mann-Whitney U test); BMI, body mass index; VO_2_max, maximal oxygen consumption.

### 2.2 Experimental protocol

**FIGURE 2:**
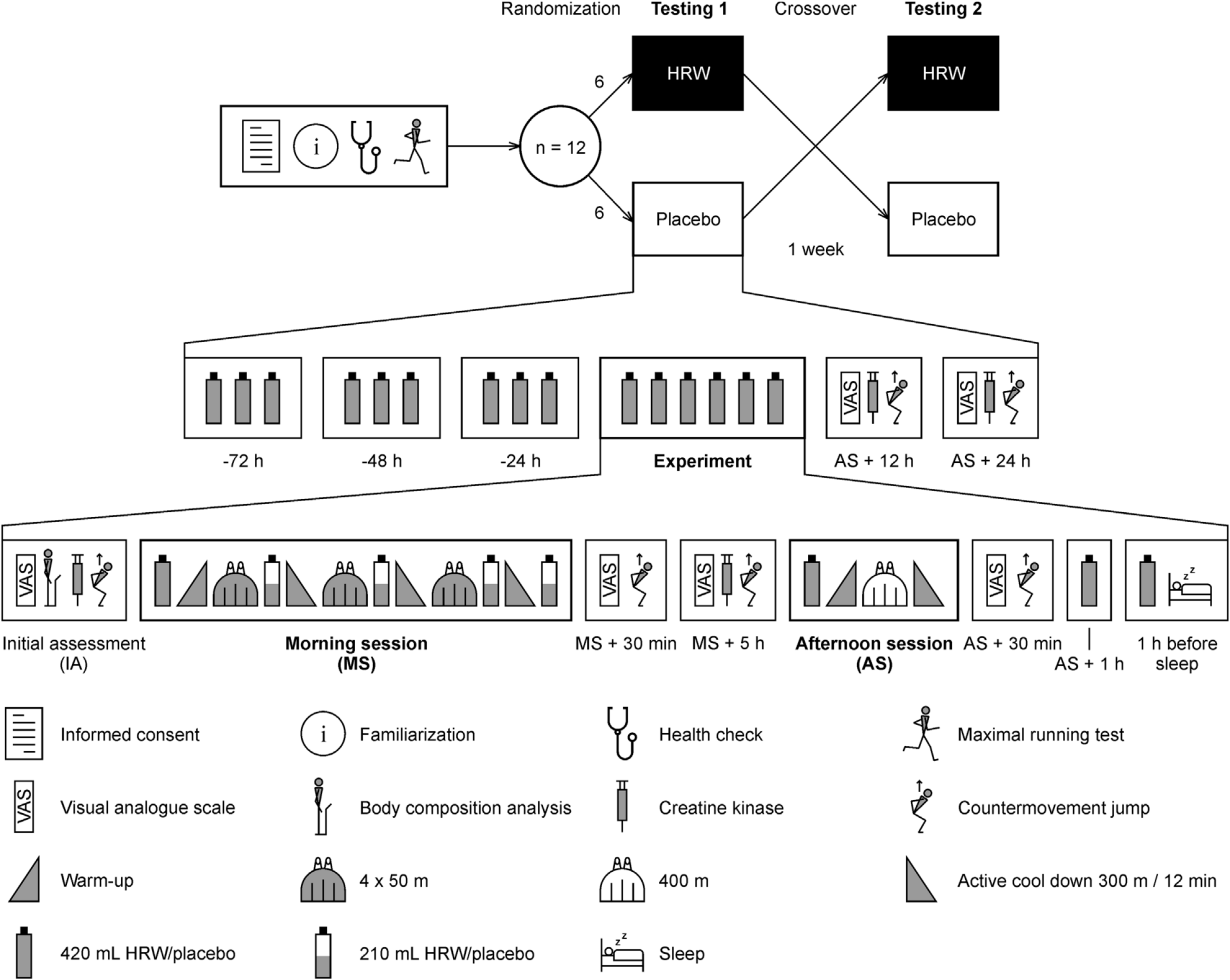
Overview of the study protocol. HRW, hydrogen-rich water.

The study had a randomized, double-blind, placebo-controlled, crossover design and was performed in a swimming pool and laboratories of the Faculty of Physical Culture, Palacký University Olomouc, Olomouc, Czech Republic. The protocol of this study was registered on ClinicalTrials.gov (NCT05799911). The experimental protocol (Figure 2) consisted of two swimming testing days, with each testing day including two swimming pool training sessions in a 25 m long indoor swimming pool and monitoring of recovery for 24 h after the second session. The initial assessment (IA) began at 7 a.m. The morning fin swimming session (MS) took place between 9 and 11 a.m. and included three high-intensity interval sets consisting of maximum 4 × 50 m swims. The time interval to complete the swim and rest was always 1 min (participants completed 50 m and rested for the remaining time up to 1 min). Between each set and after the last one, there were 300 m and 12 min of active cool down. The afternoon fin swimming session (AS) took place between 5 and 6 p.m. and included 400 m of continuous competitive performance and 300 m of active cool down. A 1,400 m warm-up was performed prior to each training unit. The washout between the tests was 1 week as was used in previous studies (Botek et al., 2021; 2022a; Valenta et al., 2022).

### 2.3 HRW and placebo characteristics and administration

The HRW (Aquastamina-R, Nutristamina, Ostrava, Czech Republic) and placebo (Aquastamina-H_2_ free, Nutristamina, Ostrava, Czech Republic) were contained within 420 mL plastic aluminum packages with a gas-tight cap. HRW/placebo characteristics were as follows: pH 7.9/7.7, oxidation-reduction potential −652/+170 mV, temperature 20/20°C (pH/ORP/Temperature-meter AD14, Adwa Instruments, Szeged, Hungary), and dissolved H_2_ concentration 0.9/0.0 ppm (H2Blue reagent, H2 Sciences, Henderson, NV, USA). Because H_2_ is colorless, odorless, and tasteless (Nicolson et al., 2016), it was not possible to distinguish HRW from placebo. The type of water (HRW/placebo) was indicated on the pack using different batch numbers. The details of the batch numbers were kept confidential by the manufacturer until the experiment was completed. Three days before testing, the participants were provided with 1,260 mL of HRW/placebo (divided into three 420 mL doses). On the day of the test, the supplementation strategy was as follows: 420 mL HRW/placebo just before MS, 210 mL after each set of swimming (morning), and after the final cool down (total 1,260 mL). Another 420 mL immediately before AS and 1 hour after AS. The last pack (420 mL) was administrated 1 hour before going to sleep. The order of HRW/placebo or placebo/HRW was determined for each participant based on a randomization table created at the beginning of the study by a technical staff member not involved in the experimental part using a random number generator (MATLAB R2020a, MathWorks, Natick, USA).

### 2.4 Visual analogue scale

The VAS was used to assess lower limb muscle pain before the exercise protocol, 30 min and 5 h after MS, and 30 min, 12 and 24 h after AS. The VAS was a horizontal 100 mm length line, marked with 0, indicating “no pain” and 100 indicating the “worst imaginable pain” (Crichton, 2001). Participants were asked to assume the position of an unweighted squat at approximately 90° of knee flexion and mark perceived pain on the 100 mm VAS (Twist and Eston, 2005; Jakeman et al., 2010).

### 2.5 Creatine kinase

CK was determined in a capillary blood sampled from the fingertip. An alcohol wipe was used to clean the fingertip and the skin was punctured with a lancet (Accu-Chek, Roche Diagnostics, Rotkreuz, Switzerland). The first drop was wiped away and the second drop was used. A Reflotron applicator with a 32 μL disposable pipette tip was used to extract a 32 μL sample of blood and place it on a CK assay strip (Reflotron CK strips, Roche Diagnostics, Rotkreuz, Switzerland). The blood sample was analyzed using a spectrophotometer (Reflotron Plus, Roche Diagnostics, Rotkreuz, Switzerland) to determine the whole blood CK activity. CK was monitored before the exercise protocol, 5 h after MS, and 12 and 24 h after AS.

### 2.6 Countermovement jump

Each participant completed an individual warm-up procedure consisting of running at an intensity of 50% of their perceived maximum speed for 3 min, ten squats, and one submaximal CMJ. After 1 minute of rest, each participant performed three single maximum effort CMJs with 30 s of rest between jumps. The starting position for the CMJ was an upright posture with the hands placed on the hips. Vertical ground reaction force was measured on two parallel force platforms (AMTI OR6-7-1,000, Advanced Mechanical Technology, Watertown, MA, USA) with a sampling frequency of 1,000 Hz. A quiet standing period of 2 s was recorded prior to the initiation of each CMJ to ensure an initial velocity of zero and to calculate the body mass. The jump height was calculated from the force-time curve using the formula published by (Vaverka et al., 2013). The average jump height calculated from three CMJs was used for statistical analysis. CMJ was measured before the exercise protocol, 30 min and 5 h after MS, and 30 min, 12 h, and 24 h after AS.

### 2.7 Statistical analysis

Power analysis was performed using G*Power version 3.1.9.7 (Faul et al., 2007). The level of statistical significance was set at α = 0.05 and power was set at 1-β = 0.80. Based on previous studies with a crossover design (Kawamura et al., 2016; Botek et al., 2021), the effect of HRW in this research was estimated to be *d*_z_ = 0.8, expressed as Cohen’s d of the difference scores (Lakens, 2013). Assuming a paired one-tailed *t*-test, the result for the required sample size was 12 participants.

Statistical analyses were performed using MATLAB R2020a with Statistics Toolbox (MathWorks, Natick, MA, USA). Data are presented as an arithmetic mean ± standard deviation. Characteristics between females and males were compared using the Mann-Whitney U test due to the small sample size of the male group. The assumption of normality for CK, VAS and CMJ was assessed using the Shapiro-Wilk test. The sphericity was assessed using the Mauchly test. The effect of HRW compared to placebo was evaluated using a paired two-tailed *t*-test for each time point. Therefore, a set of 4, 6 and 6 tests for CK, VAS, and CMJ, respectively, were used. The significance level was set at α = 0.05. The Holm-Bonferroni method (Holm, 1979) was used to control the family-wise error rate. The statistical level for the set of t-tests was adaptive and the actual level was calculated in an iterative procedure based on the number of rejected null hypotheses. The difference score between HRW and placebo (Δ = HRW - placebo) was expressed using a 95% confidence interval (CI). The effect size was evaluated using Cohen’s *d*, where the standard deviation was calculated as the pooled value of the standard deviations for males and females on a placebo. The following thresholds recommended for athletes (Hopkins et al., 2009) were used to interpret the magnitude of Cohen’s *d*: trivial 0.00–0.19, small 0.20–0.59, moderate 0.60–1.19, and large ≥1.20.

To examine the individual responses, the minimum clinically important difference (MCID) was established and the frequencies of positive responders (Δ ≥ MCID), non-responders (MCID > Δ > −MCID), and negative responders (Δ ≤ −MCID) were calculated. The significance of the odds ratio of positive/negative responders was evaluated using a chi-square test. No clinical or physiological criteria have been established to determine the MCID for CK (Machado and Willardson, 2010). Therefore, a value of 37 U.L^-1^ previously determined in the study (de Sousa Neto et al., 2022) was reused for the MCID. A systematic review by (Olsen et al., 2017) included 37 studies and reported a range of 8–40 mm for MCID in acute pain. Thresholds for differences should be lower in highly trained athletes than in recreationally trained or untrained individuals (Rhea, 2004). Therefore, in this study, we adopted the lower limit of 8 mm as the MCID for VAS. The MCID for CMJ height was recommended to be 0.2 between-subject standard deviation (Turner et al., 2015; Warr et al., 2020). The CMJ was measured three times for each occasion, therefore, it was possible to calculate the technical error according to (Hopkins, 2015). The relationship between the two difference scores was assessed using Pearson’s correlation coefficient.

## 3 Results

Raw data are available in the Supplementary Material. The characteristics of participants are presented in Table 1. As expected, there were significant differences in body height, body mass, body fat, and maximal oxygen consumption between females and males. Each participant received 2.8 mmol of H_2_ dissolved in a total of 6,300 mL of HRW during the experimental protocol. The dose relative to body mass was 46.6 ± 6.1 μmol kg^−1^ for females and 36.6 ± 4.5 μmol kg^−1^ for males. The difference in relative dose was statistically significant (*p* = 0.012), which was caused by the statistically significant difference in body mass (*p* = 0.012, Table 1).

**FIGURE 3:**
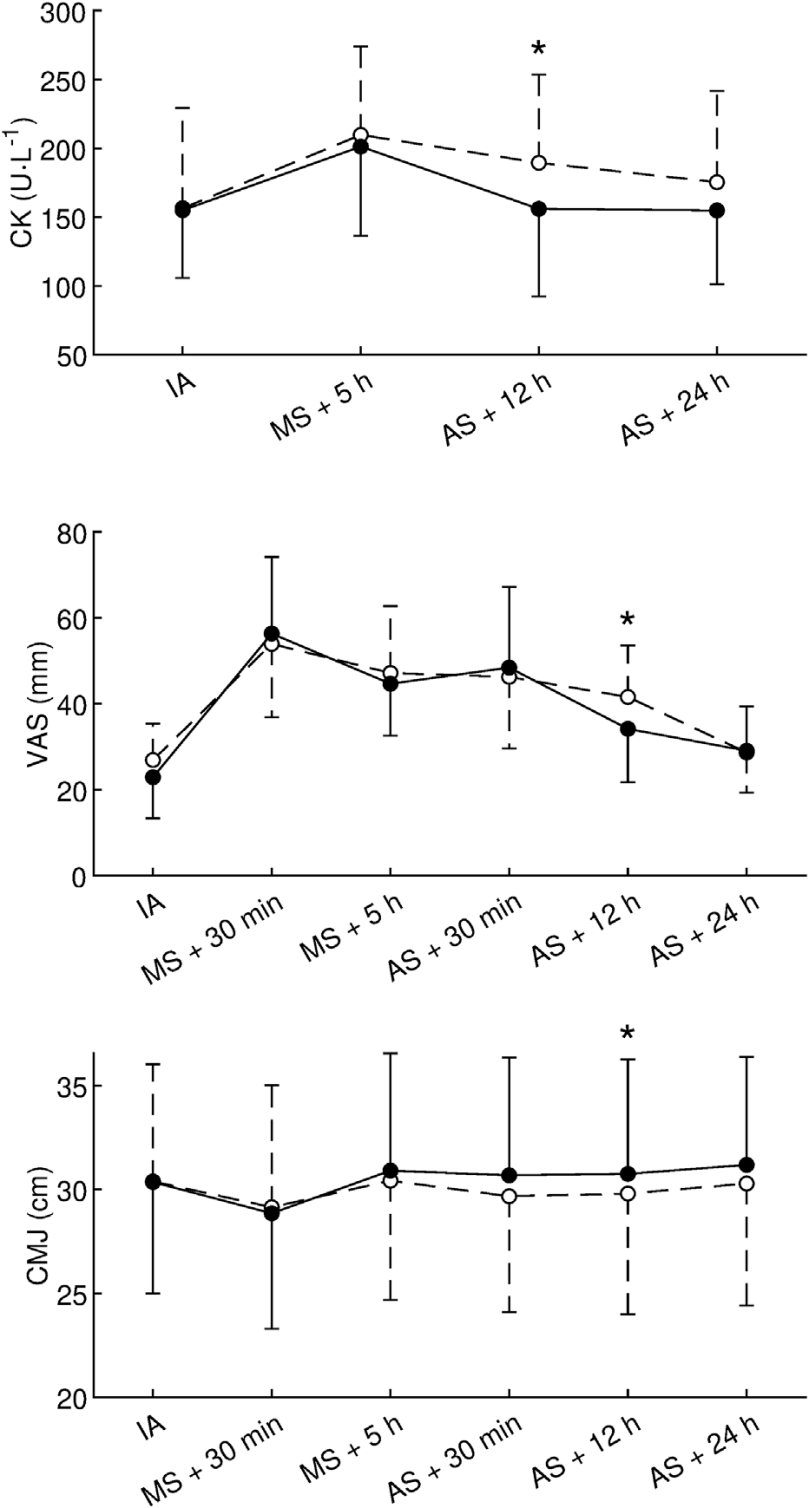
Effect of hydrogen-rich water on creatine kinase, visual analogue scale, and countermovement jump. CK, creatine kinase; VAS, visual analog scale used to assess muscle soreness; CMJ, countermovement jump height; IA, initial assessment; MS, the morning session; AS, the afternoon session; •, hydrogen-rich water; ○, placebo; ⋆, statistically significant (*p* < 0.05, paired *t*-test) difference between hydrogen-rich water and placebo. Values are presented as the mean and standard deviation.

The effects of HRW on CK, VAS, and CMJ are given in Table 2; Figure 3. However, since the sphericity was rejected for CK (*p* < 0.001), VAS (*p* = 0.002), and CMJ (*p* = 0.001), individual paired t-tests were used to evaluate the effects of HRW compared to placebo instead of repeated measures analysis of variance. HRW decreased CK at all times compared to placebo, but a statistically significant decrease was found only 12 h after AS (HRW: 156 ± 63 U.L^−1^, placebo: 190 ± 64 U.L^−1^, *p* = 0.043, *d* = −0.53, small effect). In the remaining times, the decreases were not statistically significant (all *p* ≥ 0.064, Table 2). HRW also statistically significantly reduced VAS 12 h after AS (HRW: 34 ± 12 mm, placebo: 42 ± 12 mm, *p* = 0.045, *d* = −0.74, moderate effect). However, in the remaining times, the changes were not statistically significant (all *p* ≥ 0.13, Table 2). HRW significantly improved CMJ 12 h after AS (HRW: 30.7 ± 5.5 cm, placebo: 29.8 ± 5.8 cm, *p* = 0.014, *d* = 0.21, small effect). In the remaining times, the changes were not statistically significant (*p* ≥ 0.041, Table 2). Note that *p* = 0.041 30 min after AS was not considered significant because, according to the Holm-Bonferroni method, in the case of two concurrent significant outcomes, both *p*-values must be less than 0.025.

**TABLE 2:** Effect of hydrogen-rich water on creatine kinase, visual analogue scale, and countermovement jump.

	HRW	Placebo	Effect of HRW	*p*	*d*
Variable	Mean ± SD	Mean ± SD	Δ (95% CI)		
CK (U.L^−1^)					
IA	155 ± 49	156 ± 73	−1 (−33 to 31)	0.92	−0.02
MS + 5 h	201 ± 65	210 ± 64	−9 (−55 to 38)	0.69	−0.14
AS + 12 h	156 ± 63	190 ± 64	−34 (−66 to −1)	0.043	−0.53
AS + 24 h	155 ± 54	175 ± 66	−21 (−43 to 1)	0.064	−0.30
VAS (mm)					
IA	23 ± 10	27 ± 8	−4 (−9 to 1)	0.13	−0.47
MS + 30 min	56 ± 18	54 ± 17	2 (−7 to 11)	0.57	0.14
MS + 5 h	45 ± 12	47 ± 16	−3 (−8 to 3)	0.33	−0.17
AS + 30 min	48 ± 19	46 ± 17	2 (−5 to 10)	0.54	0.14
AS + 12 h	34 ± 12	42 ± 12	−7 (−15 to 0)	0.045	−0.74
AS + 24 h	29 ± 10	29 ± 9	0 (−3 to 4)	0.78	0.05
CMJ (cm)					
IA	30.3 ± 5.4	30.4 ± 5.6	0.0 (−0.9 to 0.8)	0.94	−0.01
MS + 30 min	28.8 ± 5.6	29.1 ± 5.9	−0.3 (−1.0 to 0.4)	0.40	−0.06
MS + 5 h	30.9 ± 5.6	30.4 ± 5.7	0.5 (−0.5 to 1.4)	0.29	0.12
AS + 30 min	30.7 ± 5.7	29.7 ± 5.6	1.0 (0.0 to 2.0)	0.041	0.23
AS + 12 h	30.7 ± 5.5	29.8 ± 5.8	0.9 (0.2 to 1.7)	0.014	0.21
AS + 24 h	31.2 ± 5.2	30.3 ± 5.9	0.9 (0.0 to 1.8)	0.053	0.21

HRW, hydrogen-rich water; SD, standard deviation; Δ, difference between hydrogen-rich water and placebo; CI, confidence interval; *p*, statistical significance of paired *t*-test; *d*, Cohen’s d effect size; CK, creatine kinase; VAS, visual analog scale used to assess muscle soreness; CMJ, countermovement jump height; IA, initial assessment; MS, the morning session; AS, the afternoon session.

**FIGURE 4:**
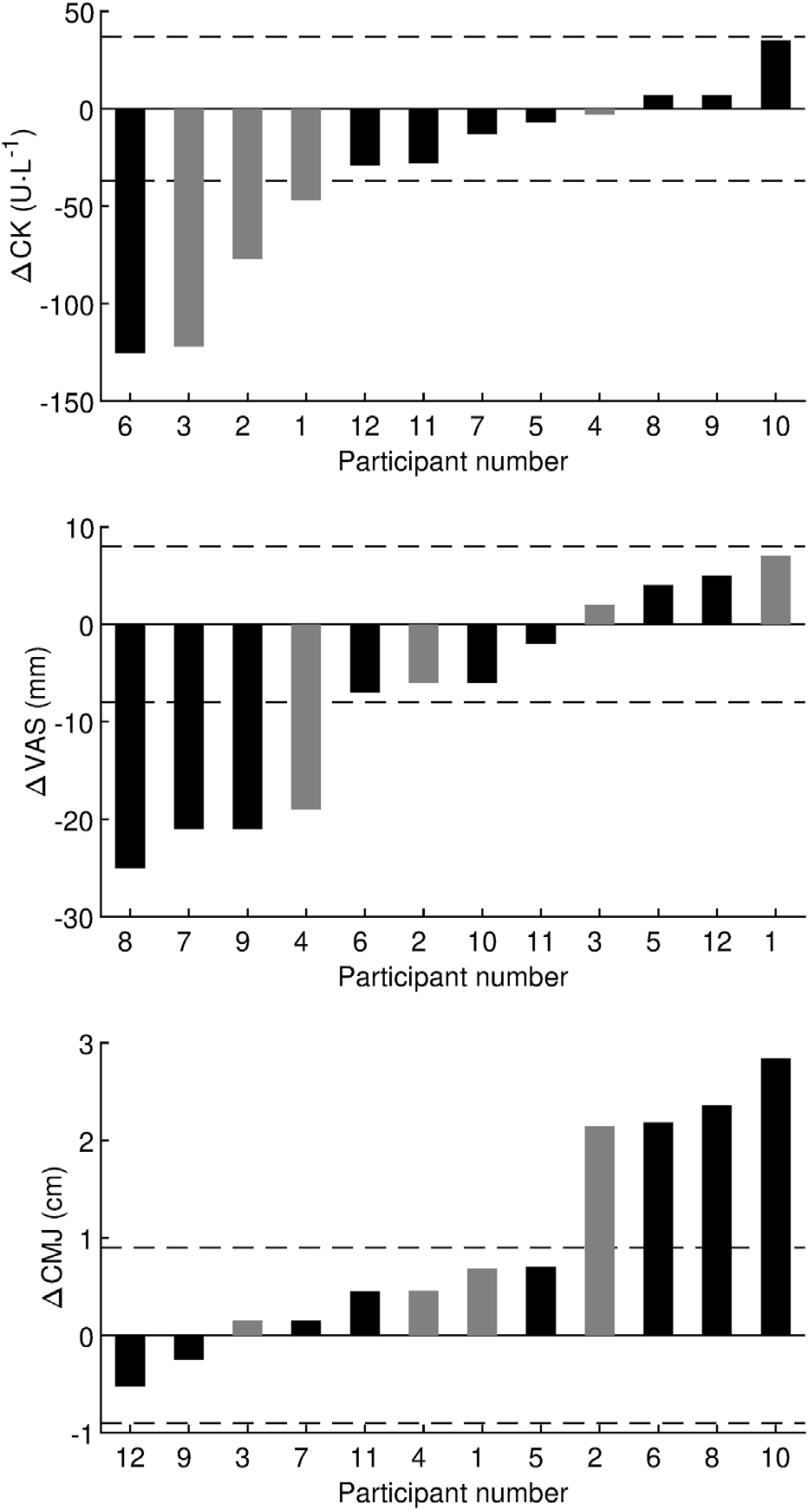
Individual responses in effect of hydrogen-rich water on creatine kinase, visual analogue scale, and countermovement jump. Δ, difference between hydrogen-rich water and placebo values obtained 12 h after the afternoon session; CK, creatine kinase; VAS, visual analog scale used to assess muscle soreness; CMJ, countermovement jump height. Black bar denotes female and gray bar denotes male. The horizontal dashed lines represent the minimum clinically important difference.

**FIGURE 5:**
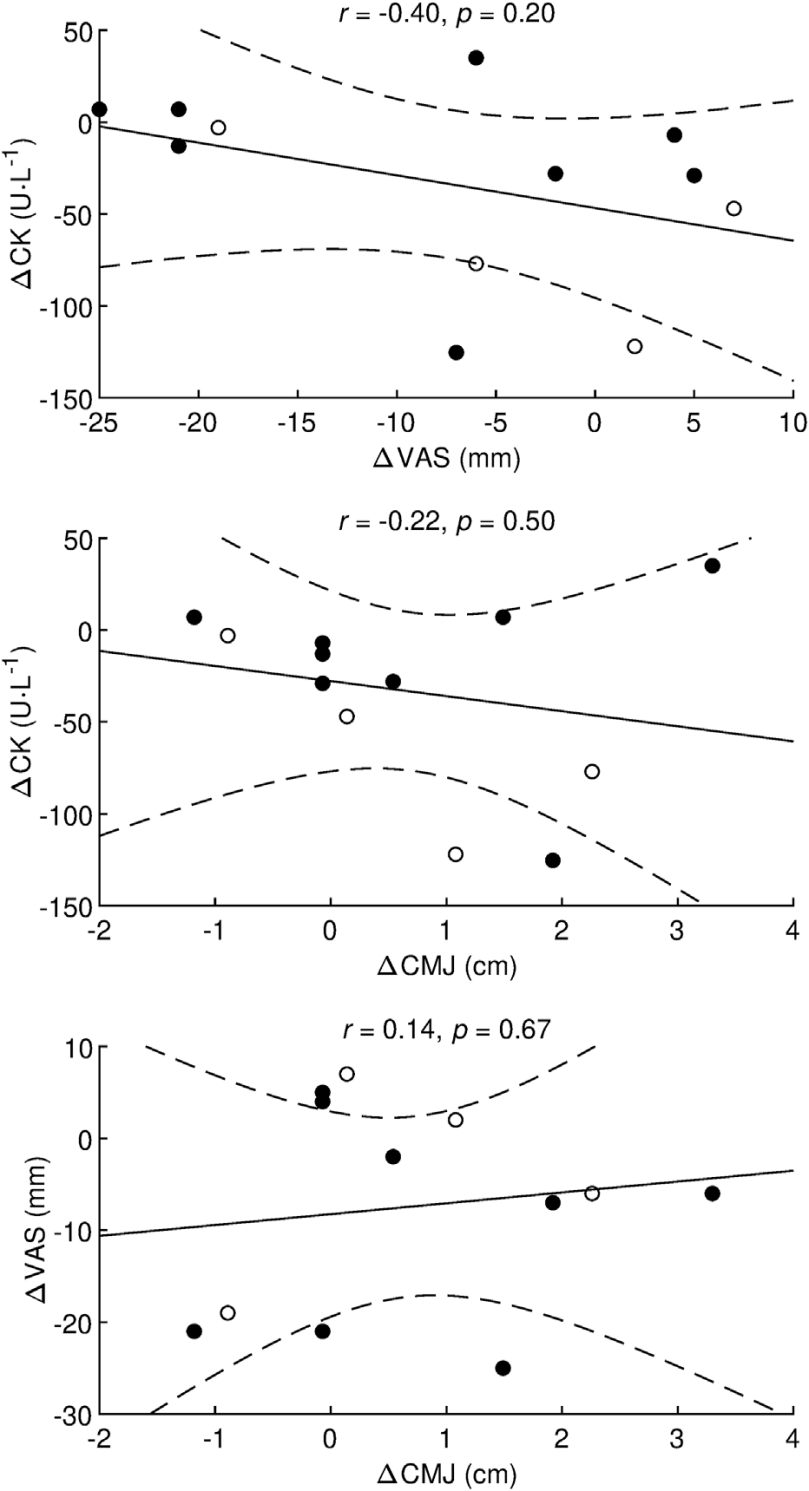
Correlation analysis between the effects of hydrogen-rich water on creatine kinase, visual analogue scale, and countermovement jump *r*, Pearson’s correlation coefficient; *p*, statistical significance of the correlation coefficient; Δ, difference between hydrogen-rich water and placebo values obtained 12 h after the afternoon session; CK, creatine kinase; VAS, visual analog scale used to assess muscle soreness; CMJ, countermovement jump height; •, females; ○, males. Dashed lines denote a 95% confidence interval.

The analysis of individual responses is shown in Figure 4. For CK 12 h after AS, four participants (3 males, 1 female) responded positively to HRW (decrease in CK greater than MCID), eight participants (1 male, seven females) did not respond, and no participants responded negatively. The odds ratio of positive/negative responders (4/0) was statistically significant (*p* = 0.046, chi-square test). For VAS 12 h after AS, four participants (1 male, three females) responded positively to HRW (decrease in VAS greater than MCID), eight participants (3 males, 5 females) did not respond, and no participants responded negatively. The odds ratio of positive/negative responders (4/0) was statistically significant (*p* = 0.046, chi-square test). Between-subject standard deviation for CMJ height 12 h after AS on placebo was 5.2 and 4.2 cm for males and females, respectively. Consequently, the pooled standard deviation was 4.5 cm and the MCID was 0.2 × 4.5 = 0.9 cm. Based on the data from this study, the technical error for CMJ height came out to be 0.5 cm. The analysis of individual responses in CMJ height 12 h after AS, showed that four participants (1 male, three females) responded positively to HRW (increase in height greater than MCID), eight participants (3 males, 5 females) did not respond, and no participants responded negatively. The odds ratio of positive/negative responders (4/0) was statistically significant (*p* = 0.046, chi-square test). The correlation analysis did not reveal a statistically significant relationship between changes in CK, VAS, and CMJ 12 h after AS (Figure 5).

## 4 Discussion

To the best of our knowledge, this is the first study to show that 4 days of HRW supplementation had a beneficial effect on post-exercise muscle recovery within 24 h, after two strenuous training sessions performed on the same day in elite fin swimmers. Specifically, the primary findings were that HRW intake compared to placebo significantly a) alleviated an indirect marker of muscle damage (reduction in capillary blood CK activity), b) reduced DOMS perception, and c) improved lower limb muscle performance at the 19 and 12 h recovery time points, after the morning HIIT and afternoon fin swimming sessions, respectively.

This study shows that CK peaked at 5 h after the morning high-intensity interval fin swimming training. The minimal rise in the mean values of CK blood activity was about 40% greater than the baseline. This finding contrasts with the usual increase in CK after exercise, which is typically ∼4-fold (Brancaccio et al., 2010). We assume that the specific type of physical task along with the water environment could explain the small increase in CK after exercise. This rise in CK may be because of the metabolic response to the physiological load of swimming rather than specific muscle damage (Lombard et al., 2012). To this situation (Mougios, 2007), previously reported that swimming involves mainly non-weight-bearing activities along with mostly concentric contractions that likely resulted in only minor EIMD, and consequently, only a small increase in the activity of blood CK could be detected. Nevertheless, a peak in CK may be because of the presence of a mild EIMD. During recovery period, blood samples exhibited a significantly lower concentration in CK with HRW compared to placebo, particularly at 19 h after morning HIIT and 12 h after (the next morning) the second fin swimming session performed in the afternoon. It has been well documented that a progressive efflux of cytosolic CK into the blood after HIIT occurs immediately and up to several days after exercise (Leite et al., 2023). This CK efflux could result from both mechanical muscle damage and indirectly through increased permeability of muscle cell membranes (Pyne, 1994; Brancaccio et al., 2010; Peake et al., 2017). Relating to the increase in blood CK activity, a few theoretical models have been formulated explaining changes in cell membrane permeability such as an overproduction of reactive oxygen and nitrogen species during intensive exercise (Pyne, 1994; Calbet et al., 2020), or phagocyte migration into damaged muscle tissue after exercise producing both reactive oxygen and nitrogen species and pro-inflammatory cytokines (Baird et al., 2012). The increased free radical oxygen species then cause oxidative damage to cellular components such as proteins, nucleic acids, and lipids present in the cell membrane (Blake et al., 1987; Calbet et al., 2020). From an oxidative stress perspective, H_2_ has been recognized as a selective antioxidant with the capability to reduce solely the most cytotoxic oxidants–hydroxyl radical (•OH) and peroxynitrite (ONOO^−^) (Ohsawa et al., 2007; Jin et al., 2023). In this regard (Nogueira et al., 2018), previously showed, in an animal model, that 30 min of H_2_ inhalation before exercise (30 min run at 80% of maximal running velocity) significantly reduced oxidative stress based on the measurement of plasma concentration of thiobarbituric acid reactive species during 3 h of recovery. Therefore, it is tenable that 4 days of extensive H_2_ supplementation (6,300 mL of HRW) may alleviate muscle damage by altering the permeability of the muscle cell membrane by both reducing oxidative stress and balancing cellular redox in the muscle tissue. A lower blood CK immediately and at 24 h after recovery from HIIT was recently reported by (Todorovic et al., 2020) who showed that 30 min of whole-body bathing in supersaturated HRW (8 mg of H_2_ per L) is a safe procedure that attenuates muscle damage. On the other hand, some studies did not show any significant decrease in post-exercise CK (Aoki et al., 2012; Shibayama et al., 2020; Botek et al., 2021) after H_2_ administration. Although the most effective way to administer H_2_, as well as what the optimal dose is, has been discussed in sports medicine for a long time (Kawamura et al., 2020), repetitive HRW consumption, particularly before, immediately after exercise, and in later phases of the post-exercise recovery may be considered a promising approach for alleviating muscle damage after physically demanding exercise.

Our study further revealed that HRW intake compared with placebo caused a significant decrease of 8 mm in muscle soreness perception on the 100 mm VAS either at 19 h after morning HIIT and at 12 h after the second fin swimming session, respectively. In this regard (Olsen et al., 2017), reported that the minimum clinically important difference of 8–40 mm on a standardized scale of 100 mm, has been established in the literature. In the current study, if we take into consideration that our elite fin swimmers carried out well-planned and highly specific drills, then even a small difference in muscle pain perception the next day, may represent practically important information about the time course of recovery or readiness to perform subsequent exercise. Similarly, to these results (Botek et al., 2021), recently observed “an analgesic effect” of HRW on DOMS perception 24 h after strenuous strength training, when 1,260 mL of HRW was applied. Furthermore (Todorovic et al., 2020), reported a significant decrease in VAS compared to placebo immediately, as well as 24 h after, high-intensity eccentric exercise indicating a beneficial effect of bathing in HRW on the progression of DOMS. A reduction in VAS perception was also reported by (Kawamura et al., 2016) who demonstrated that 1-week of H_2_ bathing for 20 min, significantly reduced DOMS sensation at 24 and 48 h after a 30 min down-hill running bout at an intensity of 75% maximal oxygen consumption and a −8% slope. Though the origin of DOMS is still unclear (Armstrong, 1984; Cheung et al., 2003; Peake et al., 2017), it has been suggested that EIMD followed by cytosolic enzyme efflux, and a local inflammation response, plays an essential part in DOMS development (Peake et al., 2017; Hotfiel et al., 2018). Recently, in an animal model (Nogueira et al., 2018), found an anti-inflammatory effect of a 2% H_2_ inhaled dose, 30 min before and then 30 min during exercise at 80% of maximum running velocity. Specifically, there was a significant reduction in exercise-induced pro-inflammatory plasma cytokines, particularly tumor necrosis factor alpha, interleukin-1, and interleukin-6.

Regarding DOMS, which is associated with EIMD, a significant transient reduction in muscle strength and power (Cheung et al., 2003; Brentano and Martins Kruel, 2011) up to 48 h after exercise has been reported (Peake et al., 2017). Our results show that HRW consumption, compared with placebo, enhanced the recovery of lower limb muscle performance (vertical jump height) at 19 h after the morning HIIT and at 12 h after the second fin swimming exercise. In this study, the MCID for CMJ height was determined to be 0.9 cm. The CMJ was measured three times, and the technical error was calculated to be 0.5 cm. Therefore, the technical error was less than the MCID, which is an important requirement for detecting clinically significant change (Hopkins, 2000). The improvement in CMJ height with HRW compared to placebo was 0.9 cm which is equal to MCID. Therefore, we suggest that the recovery-enhancing effect of HRW consumption, manifested through improved CMJ height, may be considered clinically significant. Recently (Shibayama et al., 2020), assessed the effect of 60 min of hydrogen-rich gas inhalation (4% of H_2_) immediately after completion of a 30 min treadmill run at an intensity corresponding to 75% of maximal oxygen consumption followed by CMJ (5 sets × 10 repetitions). They found a correlation (*r* = −0.78, *p* < 0.01) between urinary 8-hydroxydeoxyguanosine and CMJ performance. These findings led the authors to suggest that hydrogen-rich gas inhalation during the post-exercise recovery period might improve neuromuscular performance via reducing systemic oxidative damage. A significant attenuation in the reduction (3.7%) of peak torque after 20 maximal isokinetic knee extensions, after HRW ingestion (1.5 L of HRW, H_2_ = 0.9–1.0 ppm, within 8 h before exercise), despite no changes in serum CK and markers of oxidative stress, was also reported by (Aoki et al., 2012). Although no statistically significant correlation was found between CMJ, VAS and CK in the present study, we feel that the positive effect of HRW consumption on muscle recovery and performance could be related to the combination of the antioxidant and anti-inflammatory properties of H_2_. In addition to our main findings, four positive responders were found for all variables examined, although the group of positive responders varied for each variable. No group of participants was identified that responded positively to all variables simultaneously. A more detailed analysis of positive responders is precluded by the low sample of four participants. The question of whether and how to predict positive responders to HRW supplementation remains unresolved, although some association between HRW effect and subject characteristic was found (Botek et al., 2020). Importantly, in the current study, HRW did not have a substantially negative effect as defined by MCID in any participant for CK, VAS, and CMJ. Currently, H_2_ has no known adverse effect (Cole et al., 2022; Salomez-Ihl et al., 2024) and is no on the Prohibited List (World Anti-Doping Agency, 2024), therefore HRW can be recommended as a supplement to accelerate the muscle recovery in professional athletes. However, more studies are needed to clarify on the exact mechanism of H_2_ action on the muscle recovery process after exercise.

There are some limitations in this study. Firstly, the dose of H_2_ was constant per subject for logistical reasons and was not adjusted for body mass. Secondly, molecular (e.g., immune, reactive oxygen and nitrogen species) mechanisms were not evaluated. Knowledge of molecular responses may enhance the understanding of the H_2_ mechanisms for altering DOMS perception and improvement in muscle performance during post-exercise recovery. From practical standpoint, the limited ability to control the adherence of elite athletes to follow all instructions regarding the daily regimen (dietary regimen, sleeping habits, and prescribed amount of tap water) within the study could be seen as a limitation. In addition, not controlling for the potential effect of the menstrual cycle phase during experiment on the primary outcomes could be also considered a limitation.

## 5 Conclusion

Four days of HRW supplementation represents a promising hydration strategy for enhancing recovery after two strenuous swimming training sessions performed on the same day in elite fin swimmers. Lower limb muscle power performance and markers of EIMD, including capillary blood CK activity and DOMS were all enhanced by HRW supplementation compared with placebo.

## Data Availability

The original contributions presented in the study are included in the article/Supplementary Material, further inquiries can be directed to the corresponding author.
